# Diverse clinical processes of 16 COVID-19 cases who concentrated infection in the same workplace in Beijing, China: An observational study

**DOI:** 10.1097/MD.0000000000023800

**Published:** 2020-12-24

**Authors:** Chi Zhang, Jing Mu, Daitao Zhang, Jiawen Li, He Wang, Yunv Jin, Yan Han, Haiyang Li, Chunxiao Zhang, Peng Yu, Rui Guo, Xiangfeng Dou, Yanhui Chu, Zhao Wu, Xiaoqin Dong, Guiqiang Wang, Hong Zhao

**Affiliations:** a Department of Infectious Disease, Center for Liver Disease, Peking University First Hospital, No.8 Xishiku Street, Xicheng District, Beijing, China; b Department of Neurology, Beijing Hui people Hospital; c Beijing Center for Disease Prevention and Control; d Department of Radiology, Peking University First Hospital, No. 8 Xishiku Street, Xicheng District, Beijing, China; e Department of Respiratory Disease; f Department of Emergency Medicine, Beijing Hui people Hospital; g Department of Radiology, Beijing Hui people Hospital; h Beijing Center for Preventive Medical Research, Beijing, China; i Xicheng District, Beijing Center for Disease Prevention and Control; j Department and Institute of Infectious Disease, Tongji Hospital, Tongji Medical College, Huazhong University of Science and Technology, Wuhan; k Peking University International Hospital, Beijing, China.

**Keywords:** concentrated infection, COVID-19, diverse clinical processes, prevention, SARS-CoV-2

## Abstract

Since December 2019, an outbreak of COVID-19 sweeping the world. Understanding the clinical and SARS-CoV-2 dynamic changes of mild and ordinary patients of COVID-19, so as to provide basis for the prevention and control of COVID-19.

On February 1st, 2020, 16 SARS-CoV-2 RNA positive patients diagnosed in the same site in Beijing. The patients symptoms, signs, medication, and SARS-CoV-2 results were recorded.

Of the 16 patients, 12 were female. Although they were infected at the same time in the same workplace, their clinical processes were very different and can be roughly divided into three different types: persistent sputum positive, persistent stool positive and persistent both positive. In 7 patients with mild clinical manifestations, the median days of SARS-CoV-2 RNA negative conversion in sputum samples were significantly later than those with obvious lung injury (27 days [range: 18 to 36]; 17 days, [range 6 to 25], *P* = .021). The negative conversion of SARS-CoV-2 RNA in stool was significant later than in sputum.

There were various clinical manifestations after SARS-CoV-2 infection, even if they were infected by the same source of infection in the same place. The presence of SARS-CoV-2 virus RNA in stool samples was longer than that in respiratory tract.

## 1 Introduction

Since the outbreak of COVID-19 at the end of 2019, a total of 90,878 people have been confirmed in China by September 19, 2020, with 4744 deaths (crude mortality 5.2%). Meanwhile, 31,200,652 confirmed cases and 960,781 deaths (crude mortality 3.1%) in worldwide.^[1]^

At present, there are many literatures about COVID-19 clinical research, but most of them focus on the diagnosis and treatment of critically ill patients.^[2–4]^ For example, Cao B et al found that advanced age, high Sequential Organ Failure Assessment (SOFA) score and D-dimer more than 1 μ g/ml were the potential risk factors for poor prognosis at an early stage.^[2]^ At present, there are few reports about cluster disease at the same workplace. Although in January 2020, Chan et al^[5]^ reported a case of family cluster disease involving 6 patients, however, in the early stage of COVID-19 illness, their mainly concern was the situation of human-to-human transmission., and there was no detailed time series data of virus dynamics changes of multi-sample types (throat swab, sputum, stool).

Therefore, it is necessary to study the clinical process, viral dynamics and virus duration of COVID-19 patients with cluster disease. So as to provide a basis for COVID-19 prevention and reduction of transmission.

## 2 Methods

### 2.1 Patients

On February 1st, 2020, a female COVID-19 patient was diagnosed by a working unit in Beijing and admitted to a designated hospital for treatment. Subsequently, the Beijing Center for Disease Control (CDC) conducted an epidemiological survey on the patients colleagues. Five of them were diagnosed because of COVID-19 suspicious symptoms such as fever and cough, so they tested positive for throat swab SARS-CoV-2. Another 50 cases of co-workers of this patient without self-discomfort were also screened, and 10 cases of SARS-CoV-2 positive were found by throat swab or sputum test. In addition, 343 people who were in close contact with the above-mentioned patients were quarantined for 14 days, and 6 complained of discomfort during the isolation period, so they were tested for SARS-CoV-2 and were later diagnosed with COVID-19. According to the arrangement of the government, 16 patients with mild illness were admitted to designated hospitals. Five patients with severe illness or systemic diseases were transferred to other hospitals for treatment. (Fig. S1; [see Fig., Supplemental Content, which illustrates the flow chart of patient enrollment]).

According to the clustering disease, clinical manifestations and SARS-CoV-2 positive, all cases were diagnosed as COVID-19 patients. The diagnostic criteria and clinical classification follow the Chinese management guideline for COVID-19 (version 7.0).^[6]^

This study has been approved by the Ethics Committee of Peking University First Hospital (2020–032) and has been registered in Chinese clinical trial registry (ChiCTR2000030096). All patients signed the informed consent form.

### 2.2 Data collection and laboratory examination

In this study, the symptoms, signs and medication of these 16 patients were recorded, the results of auxiliary examination, imaging and SARS-CoV-2 were collected, and the clinical process was summarized.

Chest imaging score: the main abnormalities of pulmonary parenchyma were ground glass shadow and consolidation. In order to quantify the degree of chest imaging lesions, a score of 0 to 72 was obtained according to the degree of parenchyma abnormality and the extent of lung involvement, of which 0 was normal.^[7–9]^ Two imaging experts scored blindly, and the dissenting imaging was evaluated and discussed by the third expert.

The detection of SARS-CoV-2 RNA was carried out by Beijing CDC. The samples were examined by an N-gene-specific quantitative RT-PCR assay, as described elsewhere.^[10]^ The Ct (cycle threshold) value is negatively correlated with SARS-CoV-2 RNA, and if it is more than 38, it will be negative.^[11,12]^

### 2.3 Statistical analysis

Quantitative variables were expressed as the median (range), and categorical variables are demonstrated with number and percentage. We used the Mann–Whitney *U* test or Fishers exact test to compare differences between patients who became negative in the first SARS-CoV-2 RNA reexamination and those who did not turn negative in the first SARS-CoV-2 RNA reexamination. Statistical analyses were performed using SPSS ver. 25.0 (SPSS, Chicago, IL, USA). All *P* values reported are two-sided, and a *P* value of <.05 was considered to be statistically significant.

## 3 Results

### 3.1 The clinical characteristics at onset

The median age of 16 patients enrolled in this study was 43 years old (ranging from 27 to 64 years old), including 12 females. All the 4 male patients smoked for more than 20 years, and 2 of them had hypertension at the same time. Among the female patients, only 1 case had small cell hypochromic anemia due to large menstrual volume, and the rest had no concomitant disease. Ten patients had discomfort before SARS-CoV-2 RNA test positive, 4 patients had discomfort after SARS-CoV-2 RNA test positive, and 2 patients had no discomfort all the time. There were 62.5% (10/16) fever, 56.3% (9/16) cough, 25% (4/16) sore throat, 37.5%(6/16) fatigue, 25% (4/16) diarrhea, and only 4 cases whose body temperature was higher than 38°. The heart rate and respiration of all patients were normal, and the SpO2 was more than 95%. The absolute lymphocyte count decreased in only 3 patients, and CRP increased in 1 patient. The lymphocyte count was normal in the other 2 patients with elevated CRP. Due to the limitation of conditions, the first lung imaging examination of 14 patients was performed with x-ray, and no obvious abnormality was found in 5 cases. According to the seventh edition of the scheme, 12 cases were common type, 2 cases were mild, and 2 cases were positive. Table 1 shows the clinical characteristics of 16 patients at the onset of the disease.

**Table 1:** Summary of clinical characteristics at onset.

	#1	# 2	# 3	# 4	# 5	# 6	# 7	# 8	# 9	# 10	# 11	# 12	# 13	# 14	# 15	# 16
Sex	F	F	F	F	F	F	F	M	M	F	F	F	F	M	M	F
Age	36	43	28	36	27	43	36	46	55	59	39	33	47	60	64	62
Past medical history	N	N	N	N	N	Anemia	N	HBP, Smoking	Smoking	N	DM	N	N	HBP, smoke	Smoking	N
Clinical symptom																
Onset date∗	--	--	−7	−2	−7	2	−3	−7	−7	−3	−1	−2	3	−1	1	1
Cough	N	N	N	N	Y	Y	Y	Y	Y	Y	N	N	Y	N	N	Y
Sore throat	N	N	Y	N	N	N	N	N	N	N	N	N	Y	Y	N	Y
Fatigure	N	N	N	N	N	N	Y	N	Y	Y	Y	Y	N	N	N	Y
Muscle soreness	N	N	N	N	N	N	N	N	N		Y	N	N	N	Y	N
Diarrhoea	N	N	Y	Y	N	N	Y	N	N	N	N	N	N	N	N	N
Vital signs																
T (°C)	36.5	36.4	36.8	36.5	38.0	37.4	38	38.6	38.3	36.4	37.1	37.3	38.6	37.4	36.4	39.0
BP (mm Hg)	120/78	130/70	120/70	110/60	100/65	110/65	115/70	160/98	140/83	140/80	130/78	100/70	130/78	131/79	135/83	130/80
P (/minutes)	68	70	68	70	82	78	98	92	100	76	80	90	92	72	60	98
R (/minutes)	16	16	18	16	18	16	18	18	22	16	18	19	20	16	14	28
SpO_2_ (%)	95	96	98	99	98	100	98	96	95	97	99	97	98	97	100	94
Blood routine and CRP																
WBC (×10^9^/L)	3.11	5.67	7.14	4.34	4.47	3.46	2.29	5.31	2.25	4.71	4.12	4.01	6.09	6.28	5.32	4.56
Neutrophil (%)	54.2	56.7	75.4	47.8	64.4	48.7	53.6	57.2	57.6	64	58.5	37.1	64.3	64	68.8	50.9
Lymphocyte (%)	29.8	34.3	20	37.1	27.3	41.6	35.8	40.7	36.5	28.8	30.1	53.8	26.9	19.5	23.5	44.1
Lymphocyte count (×10^9^/L)	0.93	1.94	1.43	1.61	1.22	1.44	0.82	2.46	0.82	1.36	1.24	2.16	1.64	1.22	1.25	2.01
HBG (g/L)	142	130	136	122	144	85	146	148	143	110	129	134	137	147	140	131
PLT (×10^9^/L)	136	164	277	108	134	257	129	190	109	195	222	171	233	189	217	330
CRP (mg/L)	6.07	3.65	0.09	0.22	0.91	1.26	3.95	7.6	17.97	9.91	21.46	1.94	3.17	0.73	1.35	19.06
Image scoring	0	2	0	0	2	0	4	13	12	13	10	12	12	4	0	6
SARS-CoV-2 PCR test																
Test date	Feb 1	Feb 1	Feb 1	Feb 1	Feb 1	Feb 1	Feb 4	Feb 1	Feb 4	Feb 7	Feb 1	Feb 17	Feb 1	Feb 8	Feb 1	Feb 1
Sample type	sputum	sputum	swab	swab	swab	sputum	swab	swab	swab	sputum	swab	sputum	sputum	swab	swab	swab
Ct value	22.0	26.0	30.4	24.0	22.0	22.1	33.8	34.0	34.0	30.3	24.0	30.0	22.0	23.0	29.0	32.2
Clinical Types	Infected	Moderate	Mild	Mild	Moderate	Moderate	Moderate	Moderate	Moderate	Moderate	Moderate	Moderate	Moderate	Moderate	Infected	Moderate

### 3.2 Clinical course and outcome

One patient (#16) was transferred to another hospital on the 11th day after admission because of a gradual increase in body temperature and a progressive increase in chest tightness and belching. Another 64-year-old patient (# 15) was transferred to another designated hospital on the 17th day in accordance with the relevant regulations of Beijing CDC. The remaining 14 patients were cured.

The clinical symptoms of the patients were relieved within 10 days except cough. There were few abnormalities in vital signs and blood routine monitoring. CT scan was performed in all patients on 9 February, among the 5 patients with normal imaging (Image scoring = 0) on admission, only 2 patients remained normal all the time. The pulmonary imaging changes of all patients were significantly absorbed within 20 days. All patients received interferon atomization, and some of them were treated with chloroquine phosphate (# 12) or Lopinavir and Ritonavir (#6 and #9) for a short time.

**Figure 1:**
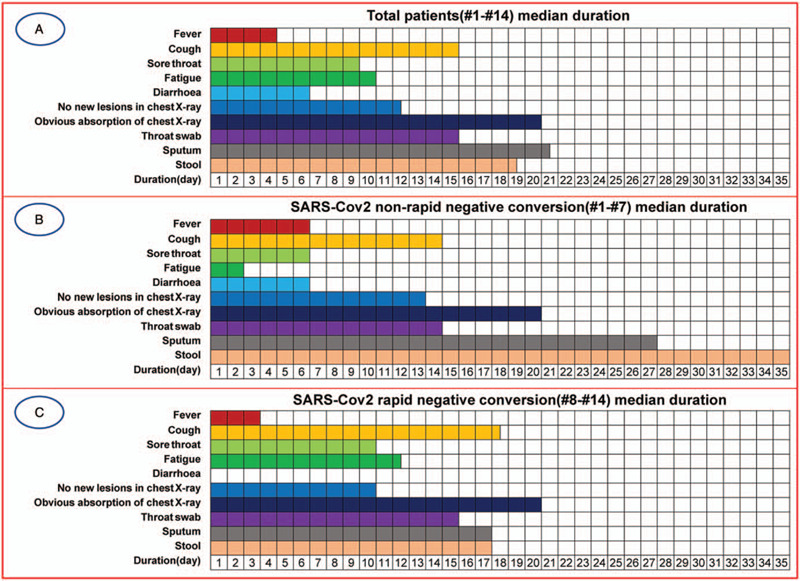
Clinical course and SARS-CoV-2 RNA test results in 3 types of samples of patients. (A) Total patients median duration, (B) SARS-CoV-2 non-rapid negative conversion median duration, (C) SARS-CoV-2 rapid negative conversion median duration.

The negative conversion of SARS-CoV-2 RNA in pharyngeal swabs of all patients was at least 2 days earlier than that of sputum, and sputum turned negative on 20.5 days (median days) after onset. At the same time, it was found that all 7 patients from # 8 to # 14 were negative for SARS-CoV-2 RNA when they were tested for the first time. The sputum negative conversion in patients from # 8 to # 14 was significantly earlier than that in patients from # 1 to # 7 (17 vs 27 *P* = .021), and stool negative conversion was also earlier. SARS-CoV-2 RNA in stool of patients # 1 and # 5 was still positive on the 39th and 46th day after onset, although the Ct values were all more than 30 (Table 2). Figure 1A shows the clinical experience of all patients and the persistence of different sample types of SARS-CoV-2 RNA, while Figure 1B and C show the clinical experience of patients with non-negative (#1 to #7) and negative (#8 to #14) conversion for the first time, respectively.

**Table 2:** Comparison of days required for negative conversion of SARS-CoV-2 RNA among three types of samples.

Sample types	#1–14	#1–7	#8–14	*P* value
Swab (days)	15.0 (6.0–29.0)	16.5 (14.0–29.0)	15.0 (6.0–21.0)	.806
sputum (days)	20.5 (6.0–36.0)	27.0 (18.0–36.0)	17.0 (6.0–25.0)	.021
Stool (days)	18.5 (6.0–46.0)	35.0 (16.0–46.0)	17.0 (6.0–23.0)	.083

Although the condition of the patients was mild, the clinical manifestations were also different. According to the SARS-CoV-2 RNA test, COVID-19 patients can be divided into 3 different types: persistent sputum positive; persistent stool positive; persistent sputum and stool positive.

**Figure 2:**
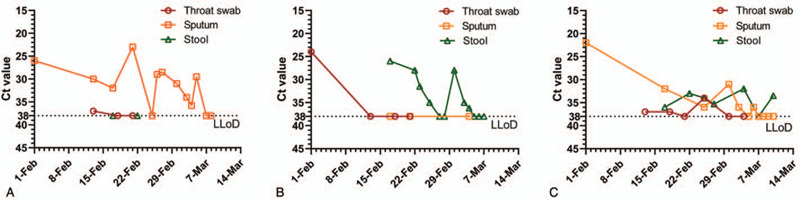
Line chart of SARS-CoV-2 RNA test changes over time in 3 different types of patients. (A) persistent sputum positive, (B) persistent stool positive, (C) persistent sputum and stool positive.

Type 1: persistent sputum positive (# 2). The patient not only had no symptoms, but also the vital signs, SpO2, blood routine and CRP were normal all the time. Sputum Ct23.2, confirmed slight pulmonary inflammation and was completely absorbed 16 days after sputum nucleic acid was positive. However, the sputum samples remained positive for 35 days, while the CT value of fecal samples was always greater than 38 (Fig. 2A and Fig. S2 [see Fig., Supplemental Content, which illustrates the lung CT of patient #2 with mainly positive respiratory tract virus]).

Type 2: persistent stool positive (# 4). After admission, lung imaging was normal for several times (Fig. 2B and Fig. S3 [see Fig., Supplemental Content, which illustrates the lung CT of patient #4 with mainly positive digestive tract virus.]), pharynx swab Ct 24, vital signs, SpO2, blood routine and CRP were always normal. The virus RNA in pharynx swab and sputum was negative for the first time. On the first reexamination (February 17) positive (Ct = 26) continued until the 35th day of the course of the disease turned negative. The presence of this case suggests that the digestive tract may be the only or major lesion of SARS-CoV-2 in some patients.

Type 3: persistent sputum and stool positive (#1). Although the total leukocyte count and lymphocyte count were decreased at admission (3110/mm^3^, 930/mm^3^), CRP was normal (6.07 mg/L), sputum Ct = 20.8, was normal, but x-ray was normal. However, on the 9th day after admission, CT revealed a small number of ground glass density foci in the lingual segment of the left upper lung. The lung image was completely absorbed on the 17th day of the course of the disease. Unexpectedly, SARS-CoV-2 RNA, could still be detected in pharyngeal swabs and sputum samples on the 25th and 36th day after the first sputum nucleic acid test was positive, while SARS-CoV-2 RNA (Fig. 2C and Fig. S4 [see Fig., Supplemental Content, which illustrates the lung CT of patient #1 with both positive for respiratory and digestive tract virus]) could still be detected in fecal samples on March 11th (39th day).

### 3.3 Dynamic changes of SARS-CoV-2 RNA quantification in different samples

**Figure 3:**
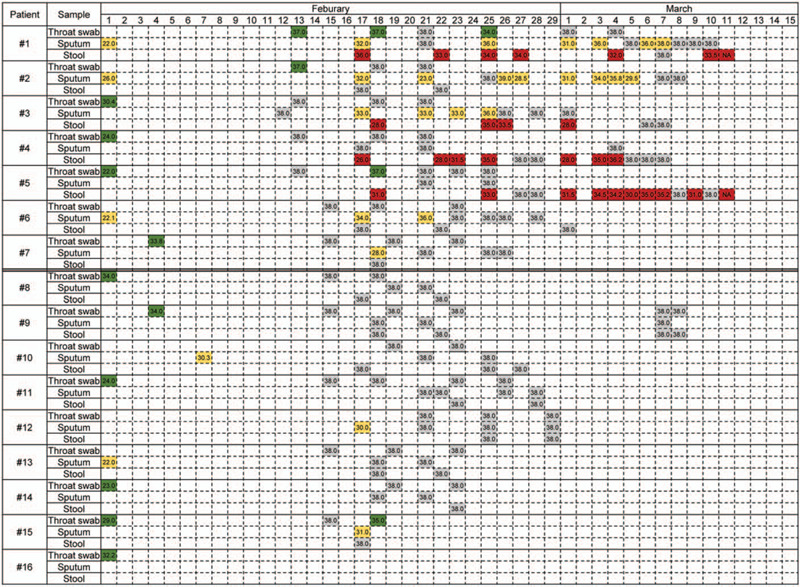
SARS-CoV-2 RNA test results in three types of samples.

After the body temperature of all patients was normal for more than 3 days, the clinical symptoms and signs basically disappeared, and the lung imaging showed that the lesions were basically absorbed, pharynx swabs, sputum and stool samples were taken to detect virus RNA, results shown in Figure 3. SARS-CoV-2 RNA in pharyngeal swabs and sputum samples of 14 patients turned negative, including those who remained asymptomatic (# 1 and # 2). The negative conversion time of SARS-CoV-2 RNA in stool was later than that of sputum and pharyngeal swab, while # 1 and # 5 were still positive for nucleic acid in stool on March 11.

It is worth noting that the Ct value was greater than 30 in the last 10 days when the positive stool persisted. There are both positive and negative tests in the same samples taken from the same patient (# 2, #5, and #6) at different times. It is suggested that the negative results of single SARS-CoV-2 RNA should be interpreted carefully. As shown in Table 3, the imaging changes of the lungs in patients # 8 to # 14 were significantly more severe at onset than those in patients # 1 to # 7 (*P* = .005).

**Table 3:** Analysis of related factors of negative nucleic acid of SARS-CoV-2 in three kinds of samples for the first review.

Variable	#1−7	#8−14	*P* value
Sex			.192
Male	0	3	
Female	7	4	
Age (year)	36 (27–43)	47 (33–60)	.025
Smoking			.192
Yes	0	3	
No	7	4	
Fever (≥37.3°C)			.592
Yes	3	5	
No	4	2	
Cough			1.000
Yes	3	4	
No	4	3	
Diarrhoea			.192
Yes	3	0	
No	4	7	
Lymphocyte count (10^9/L)			1.000
≤1.0	2	1	
>1.0	5	6	
Imaging score			.005
<10	7	1	
≥10	0	6	
Ct value	24.0 (22.0–33.8)	30.0 (22.0–34.0)	.368

## 4 Discussion

We observed 16 patients of mild symptom working in the same environment, founding that the clinical processes were different, and great attention should be paid to the infectivity of the patients with a history of exposure but mild clinical manifestations. At the same time, a variety of measures should be taken to cut off the route of transmission in controlling the epidemic situation. The digestive tract may be the only site involved in some patients. The negative result of single SARS-CoV-2 RNA detection should be treated carefully. Among the throat swab, sputum and stool samples, the persistent positive time of SARS-CoV-2 RNA detection in stool samples is the longest, accordingly, its clinical and preventive significance need more research results.

It is necessary to strengthen the management and isolation of patients of mild symptom, so as to block the transmission effectively. Existing studies pay more attention to the early warning indicators and treatment of critically ill patients, while less attention is paid to mild COVID-19 patients.^[2,13]^ Our study looked at the entire clinical process of 16 patients who were positive for viral RNA detection, showed that the mild patients still have a variety of clinical experiences. The median days of SARS-CoV-2 RNA-negative conversion in sputum samples of 7 patients with mild symptoms (27 days, range: 18–36) were significantly later than those of 7 patients with obvious clinical symptoms (17 days, 6–25 days) (*P* = .021). It is worth noting that in 2 patients without any discomfort showed RNA-positive in sputum and throat swab samples during screening (Ct value in sputum was 26 and 22 respectively), and the objective signs, change in blood routine, CRP and pulmonary imaging were also very slight. However, the positive SARS-CoV-2 RNA detection in their sputum lasted for more than 30 days. One case whose lung imaging was always normal (Ct value of throat swab 34, #3) only had diarrhea and sore throat, her objective signs and laboratory tests were basically normal, but SARS-CoV-2 RNA in sputum turned negative on the 27th day after the first examination. Those people whose amount of SARS-CoV-2 RNA arenot low but have no conscious discomfort or only mild discomfort at the time of onset, do have a greater infectious threat to the outside world than patients whose symptoms are evident. Because the formers number is large and their life and work would not be affected by the disease. Therefore, in the epidemic period, for people with a clear history of exposure, strict isolation management is needed to avoid intra-population dissemination.

The alimentary canal may be the only site involved in SARS-CoV-2.For example, there is 1 patient (#4) with mild diarrhea at onset, her throat swab Ct = 24, while chest X-ray (4 times) and CT (2 times) were all normal during the course of disease. In addition, his throat swab and sputum were negative at the first reexamination (12th and 16th day), but the Ct value of SARS-CoV-2 RNA in stool was still 26 at the first re-examination and remained positive until the 33rd day. Although we did not collect throat swabs and sputum samples in the early stage to detect RNA, and cannot completely rule out mild respiratory injury in the early stage, but several times of lung imaging indicate that there is no lung injury. So it is speculated that the main lesion site of SARS-CoV-2 is the intestinal tract. Previous studies have shown that ACE2 receptors are widely distributed in epithelial cells as well as intestinal epithelium.^[14–16]^ In particular, it should be noted that the positive duration of RNA in stool is longer. Therefore, paying attention to the isolation of patients with only mild diarrhea and their fecal treatment can help to block transmission.

The negative conversion of stool is later than that of respiratory tract specimens, so it is urgent to study its infectivity in order to formulate prevention and control measures correspondingly. Our observation found that the negative conversion order of the samples was swab, sputum and stool, if these 3 kinds of samples from the same patient were positive at the same time. The RNA in stool can still be positive for 25 days after sputum negative conversion, and the Ct value in stool can be more than 30 in the last 10 days before negative conversion. Wu YJ et al study^[17]^ of 41 people also found that the time of stool negative conversion was later than that of respiratory tract specimens. Whether SARS-CoV-2 RNA positive stool needs to be isolated or not depends on the incidence of viral particles isolated from stool SARS-CoV-2 RNA positive samples and contagious. If a live infectious virus can be isolated, strict isolation measures are needed for patients. Unfortunately, the relevant data have not been seen so far.

However, this study also has some limitations. First of all, due to the particularity of emerging infectious diseases, our sample size is small. Secondly, because COVID-19 detection needs centralized monitoring, in order to avoid unnecessary waste of medical resources, it is impossible to detect the virus RNA of pharynx swab, sputum and stool every day or frequently. This may cause the detected negative conversion time to be later than the actual patient negative conversion time.

## 5 Conclusion

In conclusion, there were various clinical manifestations after SARS-CoV-2 infection, even if they were infected by the same source of infection in the same place. The presence of SARS-CoV-2 virus RNA in stool samples was longer than that in respiratory tract. Therefore, for COVID-19 patients to lift isolation, in addition to respiratory tract SARS-CoV-2 RNA test, but also routine detection of stool SARS-CoV-2 RNA, in order to better control the spread of COVID-19.

## Acknowledgments

We thank all the patients and their families who participated in this study, as well as all the health care workers and other staff who participated in the fight against COVID-19.

## Author contributions

Chi Zhang drafted the manuscript; Jing Mu, Yunv Jin, Yan Han, Haiyang Li, Chunxiao Zhang, and Peng Yu participated in the collection and arrangement of clinical cases; Chi Zhang and Jiawen Li participated in the creation of figures and tables; Daitao Zhang, Xiangfeng Dou, and Yanhui Chu participated in the detection and review of SARS-CoV-2 RNA; He Wang and Rui Guo participated in imaging diagnosis and imaging scoring; Zhao Wu, Xiaoqin Dong, and Chi Zhang participated in the proofreading of this paper; Guiqiang Wang and Hong Zhao provided the overall principle and direction of the study.

**Conceptualization:** Jing Mu, Hong Zhao.

**Data curation:** Jing Mu, Daitao Zhang, Jiawen Li, Yunv Jin, Yan Han, Haiyang Li, Chunxiao Zhang, Peng Yu, Rui Guo.

**Funding acquisition:** Gui Qiang Wang, Hong Zhao.

**Investigation:** Daitao Zhang, Hong Zhao.

**Methodology:** Chi Zhang, He Wang, Xiangfeng Dou, Yanhui Chu.

**Project administration:** Hong Zhao.

**Resources:** Jing Mu, Yunv Jin, Yan Han, Haiyang Li, Chunxiao Zhang, Peng Yu, Rui Guo, Xiangfeng Dou.

**Software:** Chi Zhang.

**Supervision:** Gui Qiang Wang, Hong Zhao.

**Validation:** Jiawen Li, Zhao Wu, Xiaoqin Dong, Hong Zhao.

**Visualization:** Jiawen Li.

**Writing – original draft:** Chi Zhang.

**Writing – review & editing:** Chi Zhang.

## Supplementary Material

Supplemental Digital Content
