# Supplementary material for: Diverse clinical processes of 16 COVID-19 cases who concentrated infection in the same workplace in Beijing, China: An observational study
Source: Medicine (Baltimore). 2020 Dec 24;99(52):e23800. doi: 10.1097/MD.0000000000023800 (PMC7769349; doi:10.1097/MD.0000000000023800)

**Figure S1:** Flow chart of patient enrollment.

**
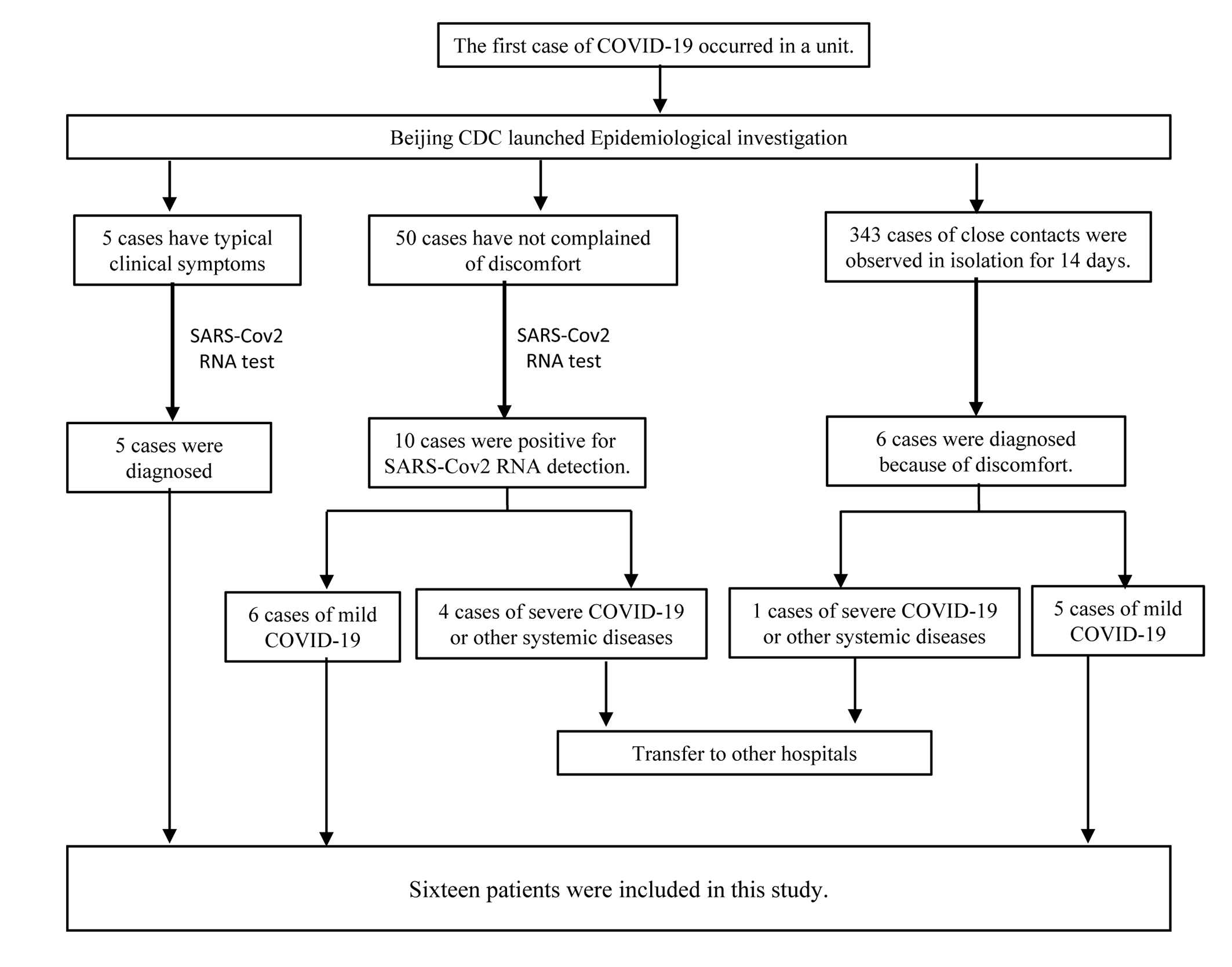
**

**Figure S2**: Mainly positive for respiratory tract SARS-CoV-2 RNA (#2).

**
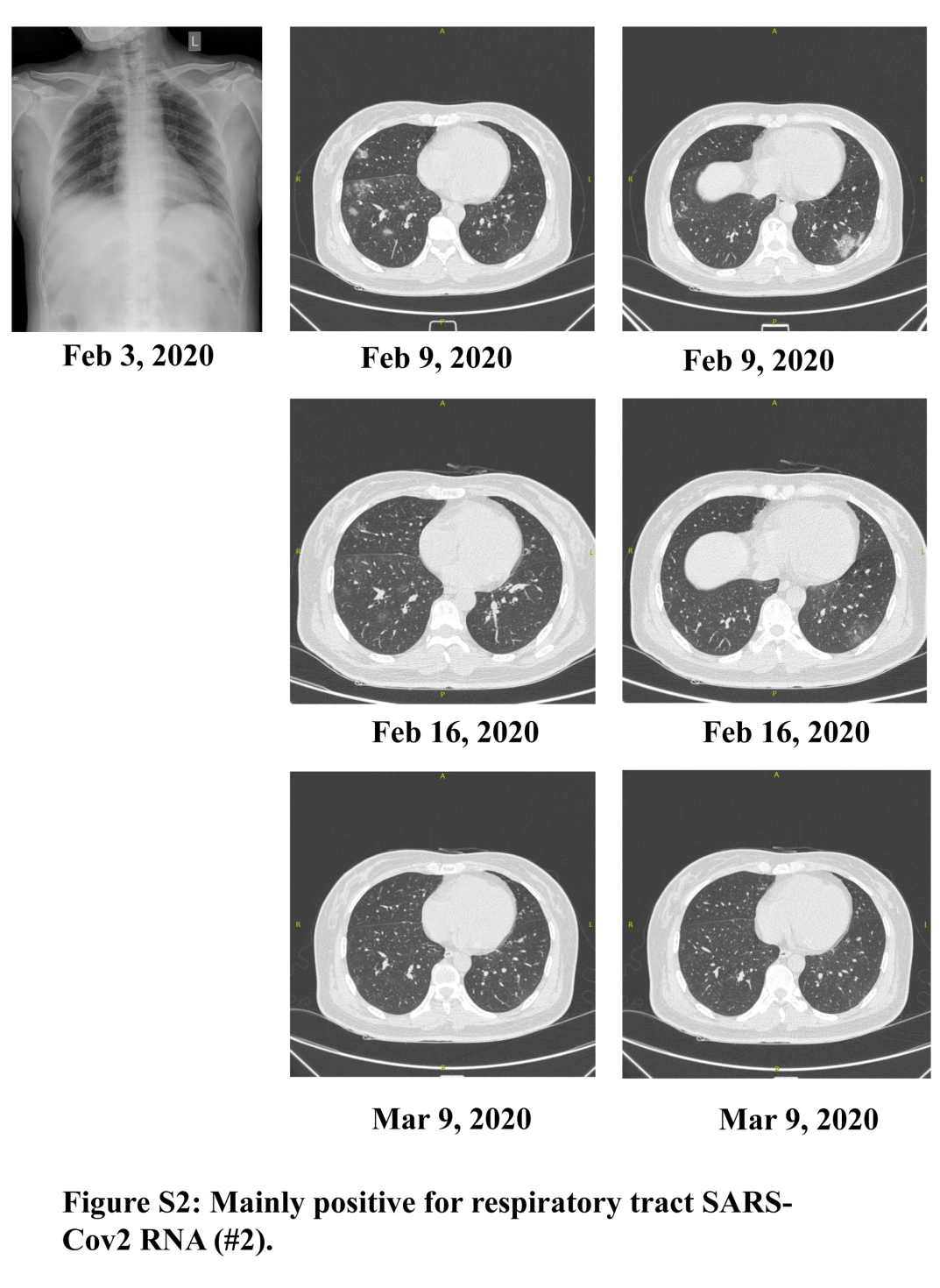
**

**Figure S3**: Mainly positive for digestive tract SARS-CoV-2 RNA (#4).


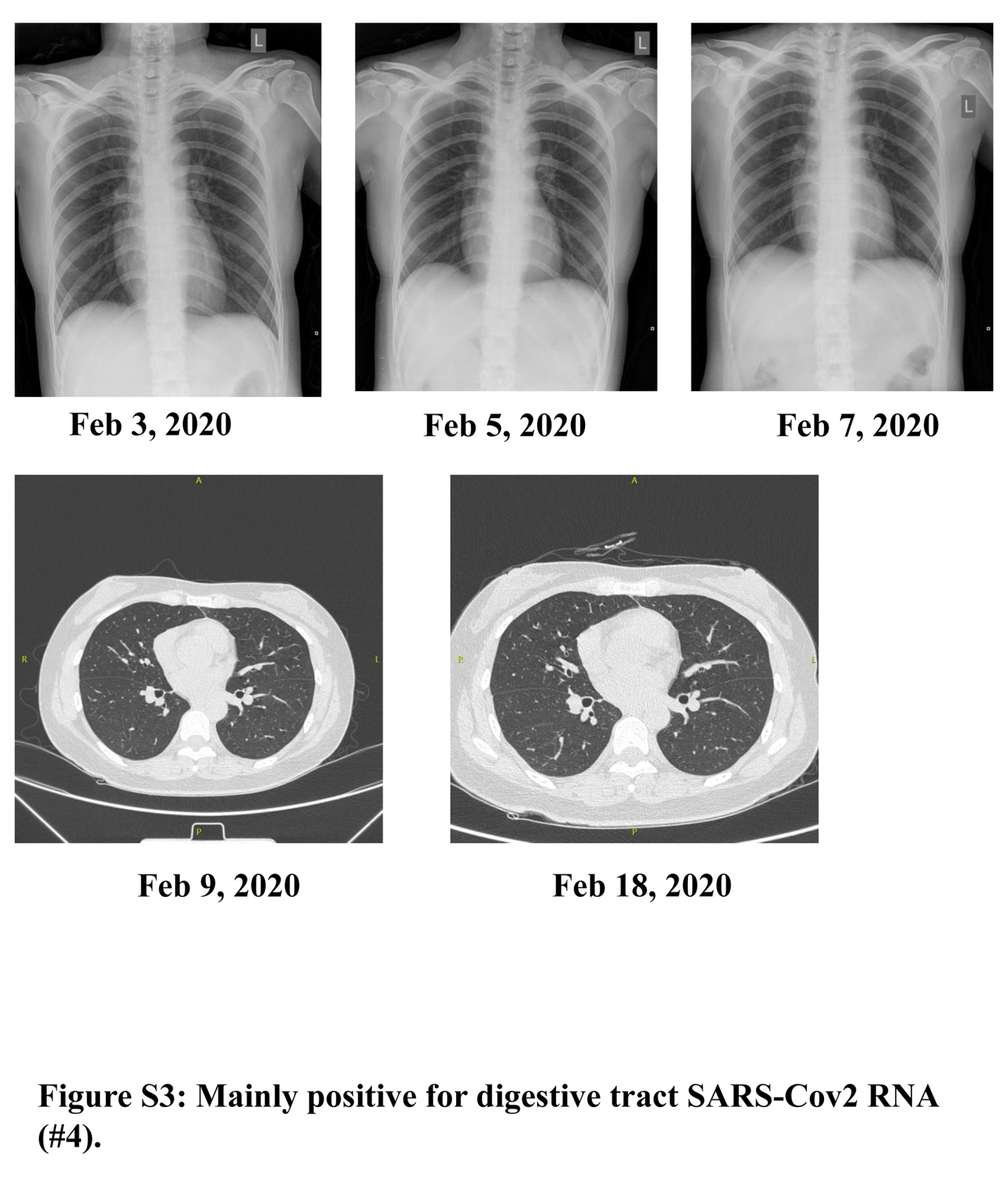


**Figure S4**: Both respiratory and digestive tract SARS-CoV-2 RNA were persistently positive. (#1).


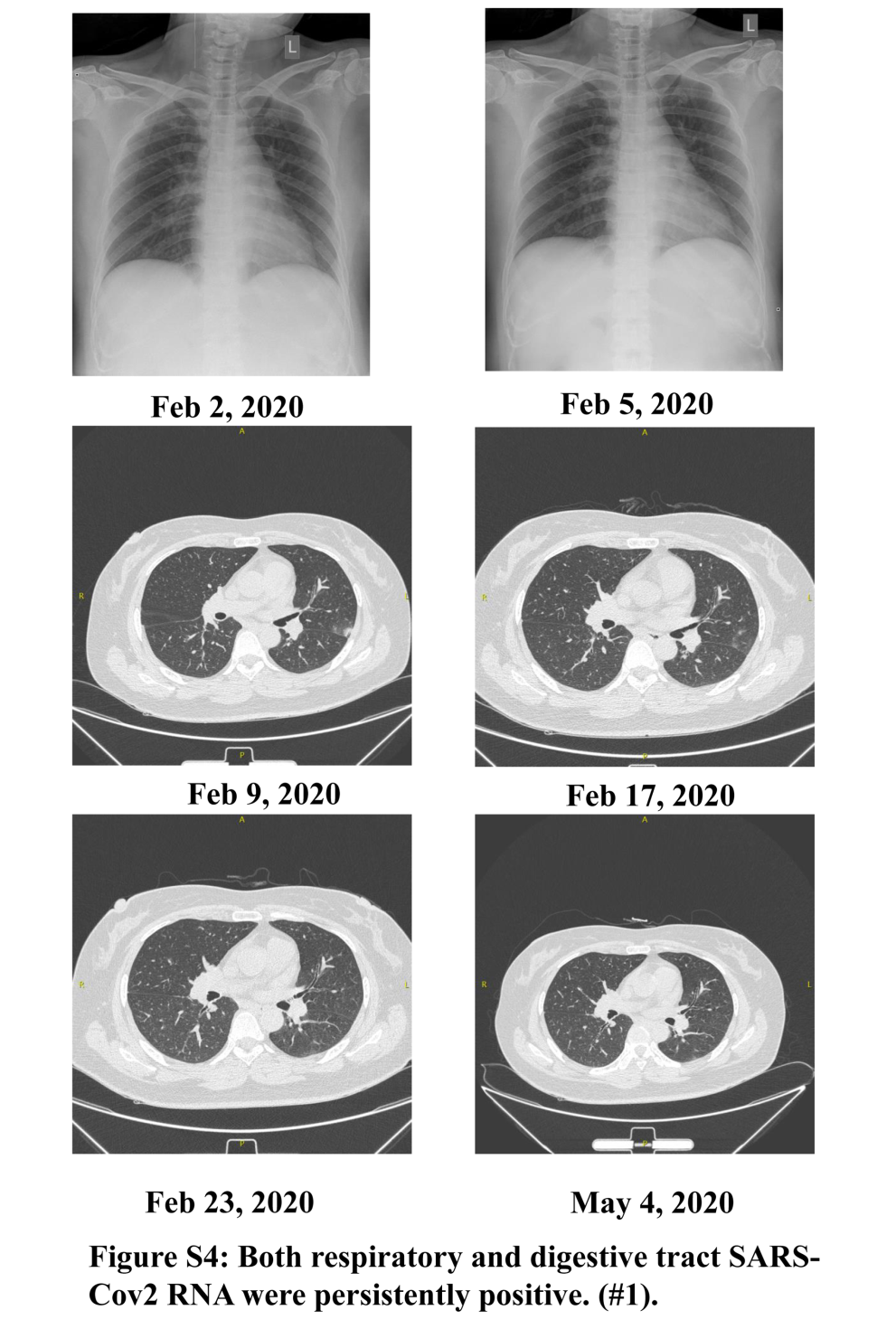

Supplement: Supplemental Digital Content [file medi-99-e23800-s001.docx]
